# *Sina* and *Sinb* genes in triticale do not determine grain hardness contrary to their orthologs *Pina* and *Pinb* in wheat

**DOI:** 10.1186/1471-2229-13-190

**Published:** 2013-11-26

**Authors:** Sebastian Gasparis, Waclaw Orczyk, Anna Nadolska-Orczyk

**Affiliations:** 1 Department of Functional Genetics, Plant Breeding and Acclimatization Institute – National Research Institute, Radzikow, 05-870 Blonie, Poland; 2 Department of Genetic Engineering, Plant Breeding and Acclimatization Institute – National Research Institute, Radzikow, 05-870 Blonie, Poland

**Keywords:** RNAi silencing, *Sin*, Triticale, Secaloindoline, Wheat, Puroindoline, *Agrobacterium*, Gene function, Orthologs

## Abstract

**Background:**

*Secaloindoline a* (*Sina*) and *secaloindoline b* (*Sinb*) genes of hexaploid triticale (x *Triticosecale* Wittmack) are orthologs of *puroindoline a* (*Pina*) and *puroindoline b* (*Pinb*) in hexaploid wheat (*Triticum aestivum* L.). It has already been proven that RNA interference (RNAi)-based silencing of *Pina* and *Pinb* genes significantly decreased the puroindoline a and puroindoline b proteins in wheat and essentially increased grain hardness (J Exp Bot 62:4025-4036, 2011). The function of *Sina* and *Sinb* in triticale was tested by means of RNAi silencing and compared to wheat.

**Results:**

Novel *Sina* and *Sinb* alleles in wild-type plants of cv. Wanad were identified and their expression profiles characterized. Alignment with wheat *Pina-D1a* and *Pinb-D1a* alleles showed 95% and 93.3% homology with *Sina* and *Sinb* coding sequences. Twenty transgenic lines transformed with two hpRNA silencing cassettes directed to silence *Sina* or *Sinb* were obtained by the *Agrobacterium*-mediated method. A significant decrease of expression of both *Sin* genes in segregating progeny of tested T_1_ lines was observed independent of the silencing cassette used. The silencing was transmitted to the T_4_ kernel generation. The relative transcript level was reduced by up to 99% in T_3_ progeny with the mean for the sublines being around 90%. Silencing of the *Sin* genes resulted in a substantial decrease of secaloindoline a and secaloindoline b content. The identity of SIN peptides was confirmed by mass spectrometry. The hardness index, measured by the SKCS (Single Kernel Characterization System) method, ranged from 22 to 56 in silent lines and from 37 to 49 in the control, and the mean values were insignificantly lower in the silent ones, proving increased softness. Additionally, the mean total seed protein content of silenced lines was about 6% lower compared with control lines. Correlation coefficients between hardness and transcript level were weakly positive.

**Conclusions:**

We documented that RNAi-based silencing of *Sin* genes resulted in significant decrease of their transcripts and the level of both secaloindoline proteins, however did not affect grain hardness. The unexpected, functional differences of *Sin* genes from triticale compared with their orthologs, *Pin* of wheat, are discussed.

## Background

Grain hardness is an important factor of cereal quality influencing the end use of common wheat [1,2]. It has a strong effect on milling properties, flour granularity and starch granule integrity. Soft wheat contains high levels of both puroindoline proteins, puroindoline a (PINA) and puroindoline b (PINB), which form friabilin [3-5]. These small proteins are unique among plant proteins, having a cysteine-rich backbone and tryptophan-rich domain [6]. Their lipid-binding properties influence dough quality [7] as well as plant resistance to pathogens [8-11]. Puroindoline-like proteins [12] in triticale are named secaloindoline a and secaloindoline b [13,14]. The function of *Sin* genes coding secaloindoline proteins is not well characterized.

Hexaploid triticale (AABBRR) is a synthetic crop developed from hybridization of durum wheat (AABB), reproducing very hard type grains with very soft type grains rye (RR). It exhibits a high level of variation for grain hardness [15]. Thus this species is very interesting to investigate the mechanism of grain hardness. As a hybrid of wheat and rye, it combines the yield potential and grain quality of wheat with the disease and environmental tolerance (soil and weather) conditions of rye. It is grown mostly for forage or fodder, although some triticale cultivars are grown for bread or are components of health food products.

*Sina* and *Sinb* genes in triticale (x *Triticosecale* Wittmack) are orthologs of *Pina* and *Pinb* in wheat (*Triticum aestivum* L.). Both species are allohexaploids containing the A, B and R or A, B and D genomes, respectively. The *Sin* genes are located in the *Hardness* locus of 5R [14,16] and the *Pin* genes in the hardness locus of 5D [3,17,18]. The well-documented, main role of *Pin* genes in wheat is to control grain hardness (reviewed in [19-21]). Specific mutation or RNAi silencing of both *Pin* genes decreased the level of puroindolines and increased grain hardness up to the level of hard *T. turgidum* var. *durum* cultivar [22]. The function of their orthologs, *Sin* genes in triticale, is expected to be similar; however, a few papers on the subject have reported contradictory data [13,14,23]. The first report on the conservation of *Pina* and *Sina* sequences reported that the homology is more than 99%, but *Sina* sequences were not found [13]. No PCR product was amplified with *Pina* specific primers in rye and triticale, but the authors documented discrete allelic variation at the *secaloindoline* loci in rye. Ramirez et al. [23] demonstrated the occurrence of a product of *Sin* genes, friabilin, but there was no correlation between the content of the starch-granule associated friabilin-like protein and grain hardness (measured by PSI). Li et al. [14] detected genes orthologous to *puroindoline b* and found that the level of starch granule-associated friabilin, which is the final product of the genes, was high in soft triticale lines and very low in hard lines. However, two newly found alleles containing substituted amino acids did not influence the phenotype. No relationship between grain hardness and *secaloindoline* alleles have been reported in rye, since all tested genotypes displayed a soft phenotype [14].

RNAi (RNA interference) technology is a great tool for silencing of native gene expression as well as for analysis of gene function and obtaining modified plants. The technology is especially useful in the case of polyploid cereals, such as wheat [22,24-27] or triticale, for which all homologous genes and their copies might be silenced. Application of RNAi technology is possible with the well-established genetic transformation method. We have already proved that for silencing of developmentally regulated genes *Agrobacterium*-mediated transformation is the method of choice, compared with the biolistic one [28]. Based on conclusions from our research and earlier documented preferences for cereal transformation [29,30], *Agrobacterium*-mediated transformation of triticale, first established by us [31], was applied in the present research.

As previously documented, the RNA-mediated silencing of one of the *Pin* genes in wheat simultaneously decreased the expression of the other, which resulted in a significant increase of grain hardness [22]. This is the first report on the RNAi silencing of their orthologs, *Sin* genes in triticale, with the aim of explaining their function. We proved the possibility of using RNAi technology in another hexaploid cereal species. Opposite to wheat, no negative correlation between very low expression level and increase of grain hardness was documented. The silencing was transmitted to the T_4_ kernel generation.

## Results

### Sequence comparison of novel *Sina* and *Sinb* alleles from cv. Wanad to other known *Sin* and to *Pina* and *Pinb* of wheat and their expression profiles

Full coding sequences of *Sin* genes of the wild plants of cv. Wanad were amplified with specific primers and sequenced. Comparison of *Sina* from cv. Wanad (deposited in GenBank under accession number KC784350) with two known *Sina* sequences from *Secale cereale* revealed 22 nucleotide substitutions and 3 nt insertions in one allele (AJ249932.1) [14] and only 4 nt substitutions in the second one (DQ269850.1) [32]. The *Sinb* sequence from cv. Wanad was deposited in GenBank under accession number KC784351. Two other accessions, AY667061.1 and AY667062.1 [13], differ to *Sinb* in one codon deletion and three or four nucleotide substitutions. The accession DQ269886.1 [32] revealed four nucleotide substitutions (Additional file 1).

Alignment/homology matrix with wheat *Pina-D1a* (DQ363911.1) and *Pinb-D1a* (DQ363913.1) alleles showed 95% and 93% homology to *Sina* (KC784350) and *Sinb* (KC784351) coding sequences of the tested triticale cultivar. Each ortholog contained at least 3 long fragments showing 100% homology: 23, 45, 53 nucleotides for *Sina* and 26, 30 and 97 nucleotides for *Sinb*. Deduced amino acid sequences showed 91.8% and 90% homology with PINA (ABD72477.1) and PINB (ABD72479.1) respectively. DNA sequence alignment of *Pina-D1a* (DQ363911.1) and *Pina-D1a* sequenced from wheat cvs. Kontesa and Torka with *Sina* of cv. Wanad revealed 22 substitutions and 3 deletions. It corresponds with 12 amino acid substitutions and one amino acid deletion. DNA sequence alignment of Pinb-D1a (DQ363913.1), Pinb-D1c sequenced from cv. Kontesa and Torka with *Sina* of cv. Wanad showed 32 substitutions and one insertion of three nucleotides (Additional file 2).

**Figure 1:**
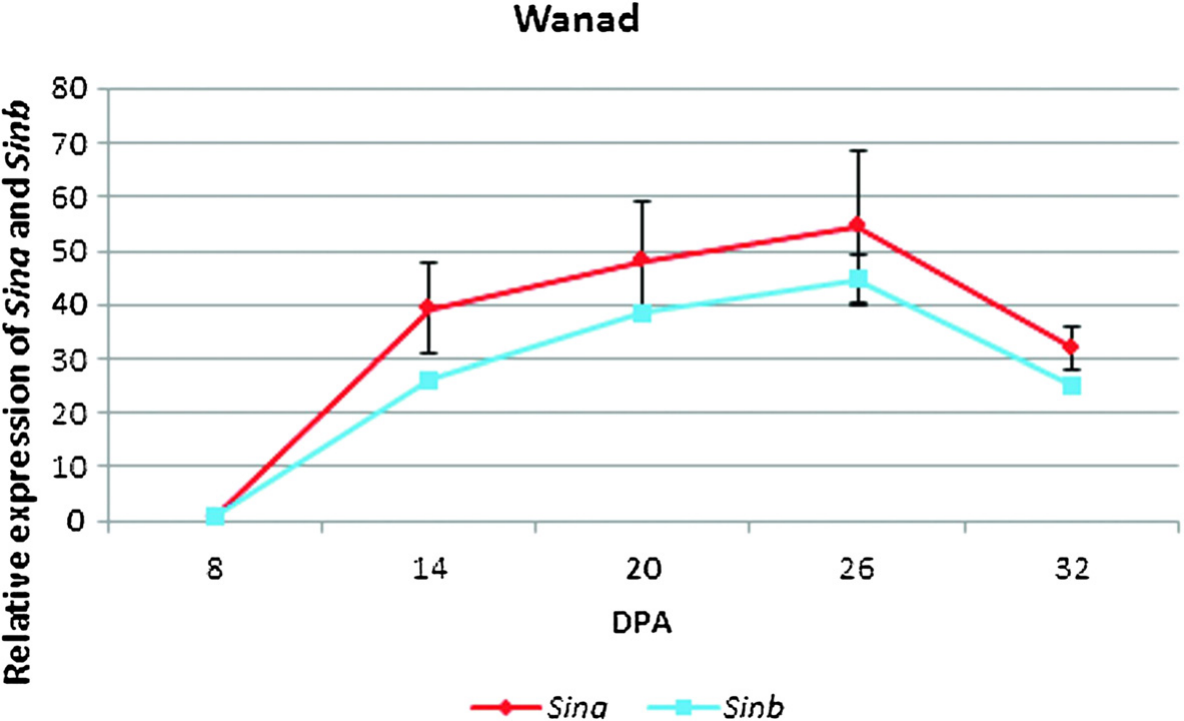
**Profiles of *Sina *** **(upper line) and *Sinb *** **(lower line) gene expression in developing spikes of the non-transgenic plants of cv. Wanad from 8**^**th **^**to 32**^**nd **^**day after pollination (DAP).**

Expression profiles of *Sin* genes in developing spikes from 8 DAP to 32 DAP of wild type plants of cv. Wanad were determined (Figure 1). The relative level of *Sina* transcript was growing up to 26 DAP and the peak was 54 times higher compared to 8 DAP. The transcript of *Sinb* started to accumulate from 8 DAP up to 26 DAP, reaching 44 times higher expression compared to 8 DAP. There was a respective decline of profiles of both genes from 26 DAP to 32 DAP. The profiles were parallel to each other, with a 20% to 30% lower transcript level of *Sinb* compared with *Sina*.

### *Agrobacterium*-mediated transformation of triticale with hpRNA type of silencing cassettes

In total, 4454 immature embryos were transformed with the (Pina), (Pinb), cotransformed with both (Pina) plus (Pinb), and with the pMCG161 empty (not containing a silencing cassette) vector (Table 1). 70 plants from 28 callus lines were selected on phosphinothricin. Out of 57 PCR tested plants 20 of them were confirmed to be transgenic with at least 3 pairs of specific primers. The transformation rate ranged from 0.00% in the case of cotransformation to 2.4% with the mean of 1.14%.

**Table 1:** Number of selected lines and plants, PCR positive plants and transformation efficiency of cultivar Wanad transformed with (Pina), (Pinb) silencing cassettes and control pMC161 (pMCG) vector

**Silencing cassette**	**Number of explants**	**Number of selected**		**PCR positive plants / transformation efficiency [%]**^**a**^
		**Lines (SD)**	**Plants (SD)**	
(Pina)	582	17 ± 0.70	39 ± 4.94	14 / 2.4
(Pinb)	1460	3 ± 1.22	17 ± 6.94	2 / 0.14
(Pina)+(Pinb)	1960	0	0	0 / 0.00
(pMCG)	452	8 ± 1.92	14 ± 3.41	4 / 0.88
Sum /mean	4454	28 **±** 1.28	70 **±** 5.1	20 / 1.14

^a^Percentage of initial explants giving rise to PCR positive plants.

### Silencing of *Sin* genes in transgenic lines through generations

**Figure 2:**
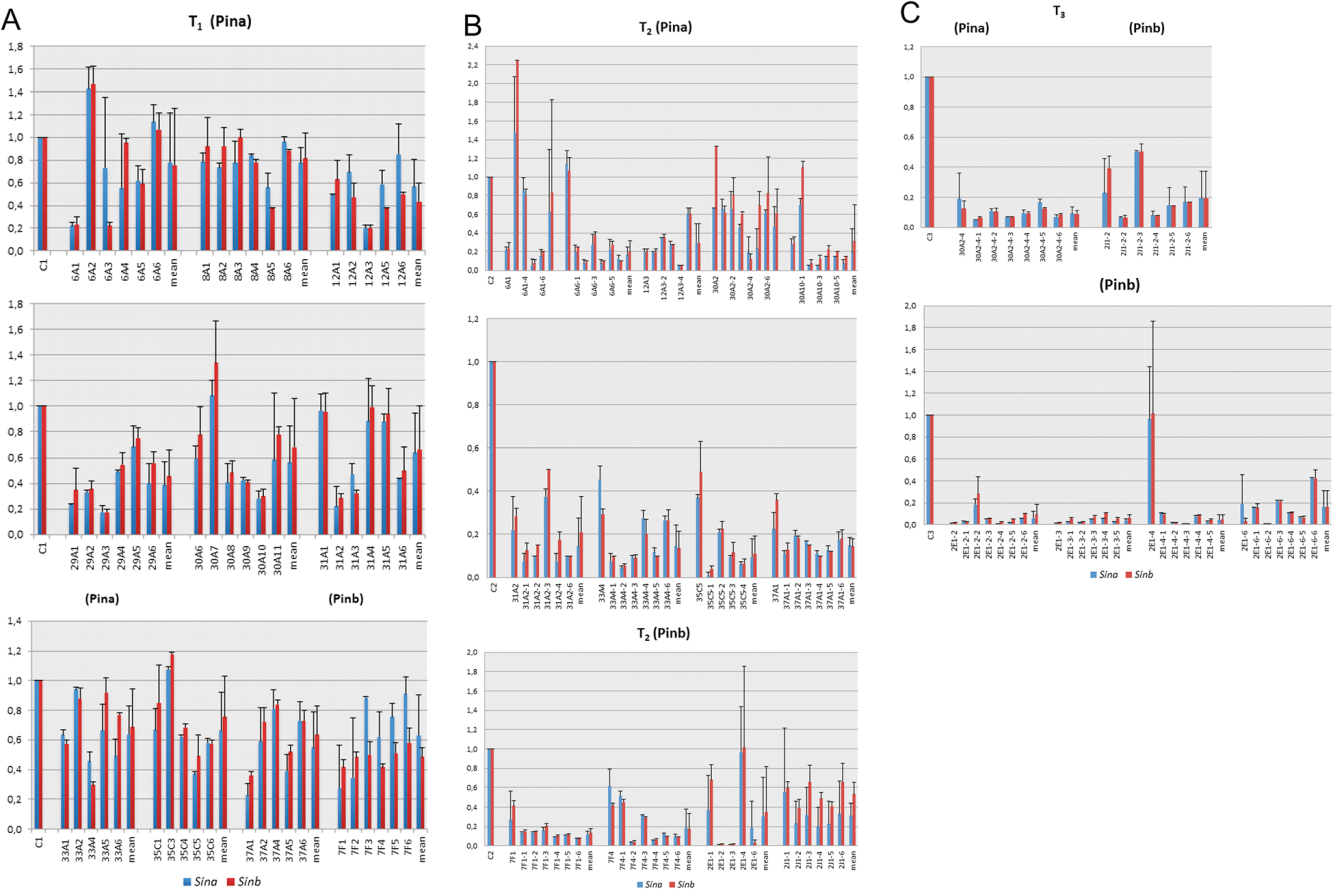
**Relative *Sina *and *Sinb *transcript levels measured by qRT-PCR in the 26 DAP spikes of the segregated progeny of T**_**1 **_**(A), T**_**2 **_**(B) and T**_**3 **_**lines (C) transformed with (Pina) or (Pinb) silencing cassettes.** C1, C2, C3 – control lines from T_1_, T_2_, and T_3_ generations respectively transformed with the empty pMCG161 vector. The first bars of T_2_**(B)** and T_3_**(C)** represent the relative values of transcript level for parent plants of the lines. The last bars are means for the respective lines.

The decrease of transcript level in silenced plants was determined in 26 DAP spikes by quantified RT-PCR. It was measured in 6 plants of each of 8 T_1_ lines transformed with the (Pina) cassette and 2 T_1_ lines transformed with the (Pinb) cassette. Relative level of expression was related to non-silenced, control plants (= 1.00), which were transformed with the empty pMCG161 vector. The transcript level for *Sina* ranged from 0.18 to 1.43 and for *Sinb* from 0.18 to 1.47 (Figure 2a). The highest level of silencing, reaching about 80% decrease of transcript, was observed in the individual plants of segregating T_1_ lines: 6A, 12A, 29A, 30A, and 31A. The lowest mean rate of both *Sin* transcripts was about 0.4 in line 29A. The levels of silencing for both *Sin* genes were similar and independent of the silencing cassette used.

Individual T_1_ plants showing in most cases the lowest transcript levels were selected to test the segregation and the level of silencing in the T_2_ generation. There were 9 lines silenced with (Pina) and four silenced with (Pinb) (Figure 2b). The range of silencing for the *Sina* gene was from 0.01 to 1.48 and for *Sinb* from 0.02 to 2.25. Most of the individual T_2_ plants were silenced and the transcript was reduced up to 99%. The level of silencing of both genes was similar, independent of the silencing cassette used. The highest differences in the levels of expression among individual plants were observed in 2E1 and 6A1 lines. Similar levels of expression were observed among plants of 2J1, 7F1 and 37A1 lines. In most T_2_ lines (9 out of 11) the mean level of transcript was lower than in the parent T_1_ plant.

As expected, individuals from lines of the T_3_ generation showed very low transcript levels for both *Sin* genes, similar among the plants of the lines (Figure 2c). It was below 0.2 in most of the tested lines as well as their T_2_ parents. Only in the case of 2E1-4 parents did the levels of *Sin* expression differ significantly from the levels of *Sin* for their progeny.

**Figure 3:**
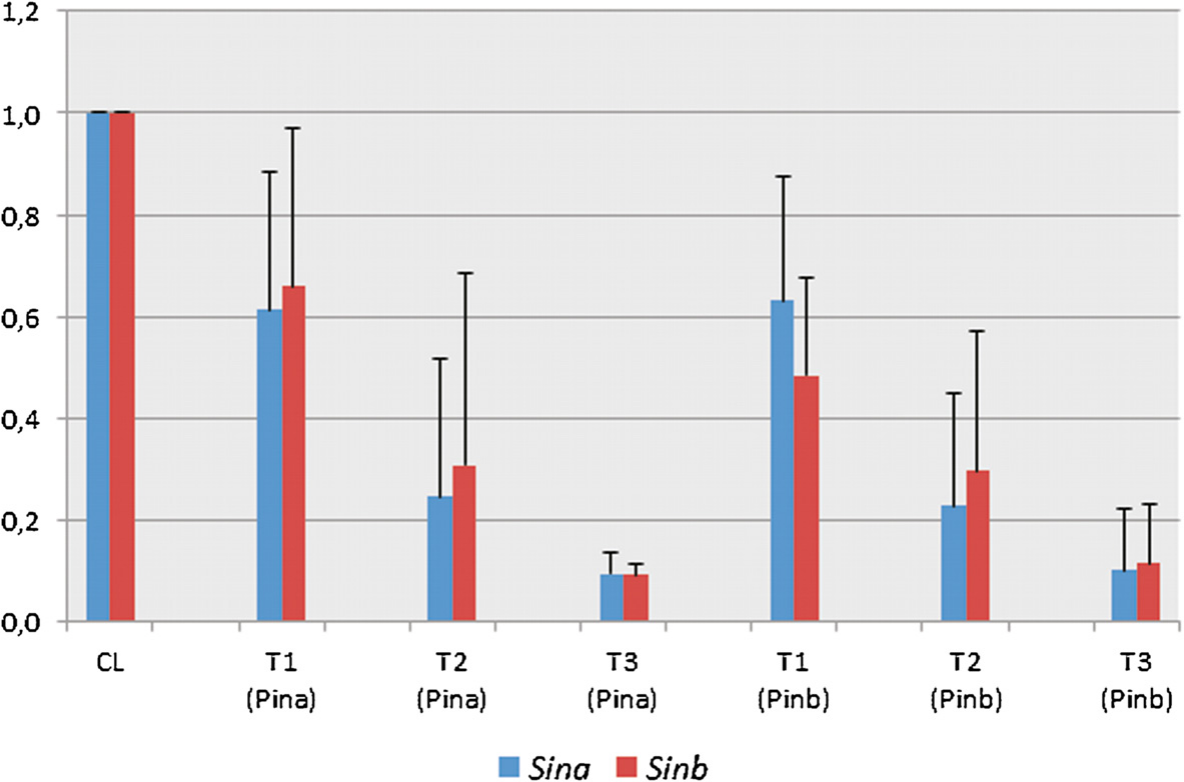
**Mean relative transcript level of *Sina *** **and *Sinb *** **for all tested individuals in T**_**1**_**, T**_**2 **_**and T**_**3 **_**generations transformed with (Pina) or (Pinb) silencing cassette.**

Data of mean relative transcript level of *Sina* and *Sinb* for all tested individuals calculated for T_1_, T_2_ and T_3_ generations (Figure 3) showed a significant decrease up to 0.1. Independent of the silencing cassette (Pina) or (Pinb), the level of silencing of *Sina* was similar to the level of silencing of *Sinb*. Transcripts of both silenced genes were lower by about 40% in T_1_ generations, about 70% to 80% in T_2_ generations, and about 90% in T_3_.

### The secaloindolines and their identification

**Figure 4:**
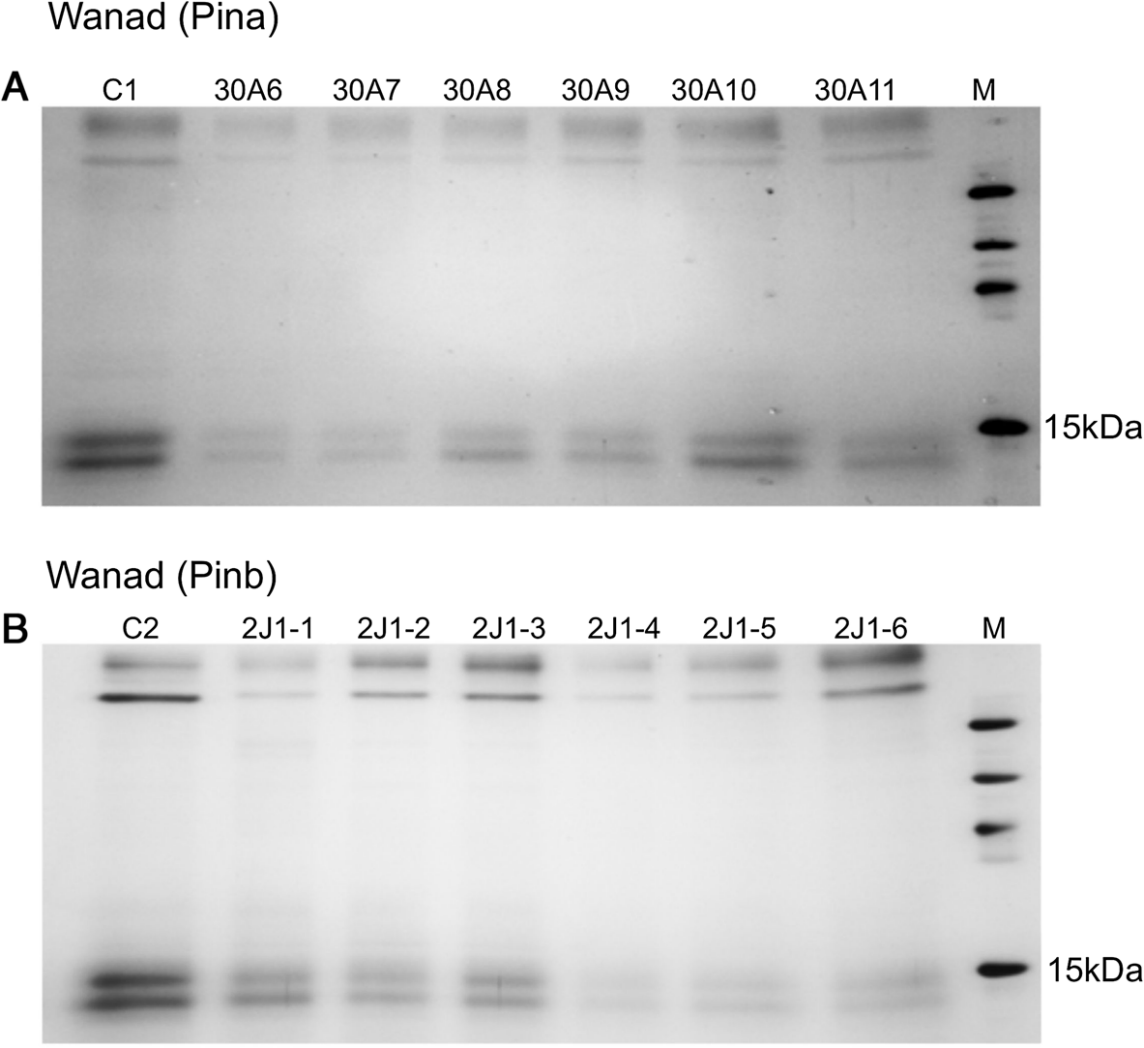
**SDS-PAGE fractionation of mature seed starch-associated proteins isolated from water washed starch from T**_**1 **_**progeny of 30A line transformed with (Pina) (A) and T**_**2 **_**progeny of 2 J1 line transformed with (Pinb): (B) C1, C2 – control lines from T**_**1 **_**and T**_**2 **_**generations transformed with the empty pMCG161 vector; M – molecular weight marker.**

Lower transcript level of the *Sin* genes was correlated with a low or very low level of secaloindoline a (SINA) and secaloindoline b (SINB) (Figure 4).

The protein contents of secaloindoline bands (<15 kDa) was identified by mass spectrometry and compared with a protein sequence database using Mascot search engine (http://www.matrixscience.com/) (Table 2) [2,32-40]. The highest scores of protein identity and number of significant matches >100 were obtained for deposited by us in GenBank SINA from triticale cv. Wanad (AGO65289.1); three others were PINA from *Aegilops sharonensis* (ABB52757.1), PINA from *Pseudoroegneria spicata* (AER62833.1) and PINB from *Secale cereale* (AAT76523.1). Two more puroindoline-like b with lower scores and significant matches were from *Dasypyrum villosum* and *Aegilops umbellulata*. The rest of proteins were dimeric alpha-amylase inhibitors from different species of *Triticum* and *Aegilops speltoides*. According to GeneBank regions 36–135 of SINA and 39–137 SINB are named as AAI_SS: Alpha-Amylase Inhibitors (AAIs) and Seed Storage (SS) Protein subfamily. They are composed of cereal-type AAIs and SS proteins [41]. Additionally both proteins contain two more sites: polypeptide binding for dimer interface order and alpha-amylase binding site order. Tryptophan-rich domain of SINA is FPVTWQWWKWWKG and differ from wild-type of *Pina-D1a* allele protein of *T. aestivum* by one amino acid (R instead of Q = arginine is changed to glutamine). The same domain of PINB is FPVTWPTRWWKG and differ from wild-type PINA by one amino acid (R instead of K = arginine is changed to lysine).

**Table 2:** **Identification of protein contents of secaloindoline bands (<15 kDa) by mass spectrometry and comparison with a protein sequence database using Mascot search engine (**http://www.matrixscience.com/**); used filters: ("Num. of significant matches">30 AND Score>42)**

**NCBI accession**	**Score**	**Mass**	**Matches Pep(sig)**	**Sequences**	**Description**	**References**
gi|515020476	15990	16803	242	8	secaloindoline a [*Triticum aestivum* x *Secale cereale*]	This paper
gi|80971579	16240	14002	225	4	puroindoline a [*Aegilops sharonensis*]	[33]
gi|355389782	14666	17031	216	4	puroindoline a [*Pseudoroegneria spicata*]	[34]
gi|50402252	3159	17220	119	14	puroindoline b [*Secale cereale*]	[2]
gi|114215762	3369	13743	59	13	dimeric alpha-amylase inhibitor [*Triticum dicoccoides*]	[35]
gi|123957	2337	18893	50	11	RecName: Full=Alpha-amylase/trypsin inhibitor CM3	[36]
gi|283137474	1284	17089	47	9	dasyoindoline-b [*Dasypyrum villosum*]	[unpublished]
gi|114215784	2683	13771	45	12	dimeric alpha-amylase inhibitor [*Triticum dicoccoides*]	[35]
gi|227809278	2498	15588	41	11	dimeric alpha-amylase inhibitor [*Triticum timopheevii* subsp. armeniacum]	[37]
gi|123958	1191	16399	41	10	RecName: Full=Alpha-amylase/trypsin inhibitor CM16	[38]
gi|114215854	2136	13948	38	9	dimeric alpha-amylase inhibitor [*Aegilops speltoides*]	[35]
gi|65993872	2248	15578	37	10	dimeric alpha-amylase inhibitor [*Triticum aestivum*]	[39]
gi|82659349	741	17357	34	8	puroindoline-b [*Aegilops umbellulata*]	[32]
gi|114215874	1655	13981	33	7	dimeric alpha-amylase inhibitor [*Aegilops speltoides*]	[35]
gi|227809121	1690	15555	31	8	dimeric alpha-amylase inhibitor [*Triticum dicoccoides*]	[37]
gi|283465827	1716	13566	31	7	putative alpha-amylase inhibitor CM2, partial [*Triticum aestivum*]	[40]

Comparison of amino acid sequences of SINA (AGO65289.1) and SINB (AGO65290.1) from triticale cv. Wanad with PINA, PINB from *T. aestivum* Chinese Spring and other puroindoline-like proteins listed in Table 2 is presented in Additional file 3. The highest percent identity (97.3% and 98.6%) was between SINA and SINB from cv. Wanad and SINA and SINB from *Secale cereale* (cv. Galma and cv. Imperial respectively). Lower data, 91.2% and 90.5% identity were between SINA and SINB from cv. Wanad and “wild type alleles” proteins PINA and PINB from *T. aestivum* cv. Chinese Spring.

### The hardness index and related traits

The grain hardness index and other characteristics, such as weight, moisture, and seed diameter, were determined using the Single Kernel Characterization System in two experiments. In the first one, 6 T_1_ plants transformed with (Pina) and 6 T_1_/T_2_ plants transformed with (Pinb) were compared with control plants (not shown). The hardness index ranged from 36.14 to 55.65 in silenced plants and from 44.4 to 49.1 in controls. Correlation coefficient between hardness and transcript level for *Sina* was 0.35 and for *Sinb* was 0.52. Hardness index was positively correlated with transcript level of *Sin* genes.

In the second one, 24 progeny plants from 12 silenced with (Pina) T_2_/T_3_ lines and 17 progeny plants from 7 silenced with (Pinb) T_2_/T_3_ lines were tested. Means for these data including total seed protein content are shown in Table 3. The hardness index ranged from 21.89 to 49.25 in silenced plants and from 37.47 to 43.48 in controls. The mean values of the hardness index were lower in two groups of transgenic lines showing significantly decreased transcript levels. The hardness index in control, not silenced lines was 40.69, in lines silenced with (Pina) it was 37.59, and in lines silenced with (Pinb) it was 34.70. The mean relative transcript levels of *Sina* and *Sinb* in the same group of lines were 1.00 and 1.00, 0.31 and 0.39, 0.12 and 0.13, respectively. Additionally, the total seed protein content of silenced lines was significantly lower compared with control lines. It was 14.62% for control and 13.70% or lower for silenced lines. There were no differences in weight or diameter of the seeds between the silenced lines and controls. The correlation coefficient between hardness and transcript level in the second group of plants for *Sina* was 0.06 and for *Sinb* was 0.11. Hardness index was weakly positively correlated with transcript level of *Sin* genes.

**Table 3:** Mean values of the grain hardness index and other characteristics (weight, moisture, diameter) determined using the Single Kernel Characterization System and total protein content in Control and lines silenced with (Pina), (Pinb)

**Lines**	***Sina *** **(SD)**	***Sinb *** **(SD)**	**Hardness (SD)**	**Weight [mg] (SD)**	**Diameter [mm] (SD**	**Protein (%) (SD)**
Control	1.00 ± 0.00	1.00 ± 0.00	40.69 ± 13.21	48.68 ± 6.06	3.13 ± 0.24	14.62 ± 0.25
(Pina)	0.31 ± 0.04	0.39 ± 0.04	37.59 ± 12.54	49.63 ± 6.48	3.13 ± 0.26	13.70 ± 0.6
(Pinb)	0.12 ± 0.01	0.13 ± 0.01	34.70 ± 11.98	47.86 ± 6.80	3.05 ± 0.27	13.64 ± 0.82

## Discussion

Alignment of newly isolated *Sina* and *Sinb* from cv. Wanad with the few known from GenBank *Sin* alleles of *S. cereale* revealed a few differences including single nucleotide substitutions and codon deletion or insertion. This comparison also showed that *Sin* alleles are more variable among themselves than *Pin* alleles, which frequently differ in only one nucleotide [19,21]. In wheat even single point mutation leading to a small differences in amino acid sequence might have an immense impact on wheat hardness [42-44]. It might change structure of the protein including conformation of tryptophan-rich domain, what have an impact on lipid-binding properties and wheat kernel hardness [reviewed in [45]. Otherwise, until now no relationship between polymorphism of *Sin* genes and grain hardness in rye or triticale has been reported [13,14,23]. However discrimination of soft and hard genotypes of triticale, which correlated with the level of starch granule-associated friabilin was presented [14]. Experimental, wild-type Wanad cultivar was classified as soft/medium-soft phenotype, accumulating high levels of SIN proteins.

Wheat *Pina-D1a* (DQ363911.1) and *Pinb-D1a* (DQ363913.1) alleles showed 95% and 93% homology with *Sina* and *Sinb* coding sequences of the tested triticale cultivar. Each ortholog contained several long, exceeding 21 nt 100% homologous fragments. This feature allowed us to apply the same, hpRNA type of silencing cassette, which has already been successfully used for silencing of *Pin* genes in wheat [22] to silence *Sin* genes in triticale. (Pina) contained the whole coding sequence of *Pina-D1a* in sense and antisense orientation; (Pinb) contained the respective *Pinb-D1a* sequences, both 447 bp long. According to the process of RNAi silencing by hpRNA constructs, short interfering RNA (siRNA), which could repress the expression of the target genes, are 21, 22 or 24 bp long (reviewed in [46,47]). The degradation of the expressed silencing construct might lead to the formation of several different siRNA, which could repress expression of the target genes.

High coding sequence homology of *Sin* with their orthologous *Pin* genes was coupled with high, more than 90% amino acid homology. Products of *Pin*, puroindoline a and puroindoline b [22], and products of *Sin* alleles, secaloindoline a and secaloindoline b, were abundant in wild type cultivars, which gave a good opportunity to test silencing of the genes by RNAi.

Temporal expression patterns of both *Sin* genes in wild type plants of triticale cv Wanad during kernel development were parallel, with slightly lower relative values of the transcript for *Sinb* and with the peak at 26 DAP for both genes. This peak of transcript accumulation indicated the best phase of kernel development to measure the level of gene silencing in silenced lines. The highest relative values of *Sina* was about 55 times higher and for *Sinb* 43 times higher compared with the first measurement at 8 DAP. Similar expression profiles were observed for *Pina* and *Pinb* in two wheat cultivars [22]. These data are also consistent with starch and gluten accumulation patterns [48,49] as it was presented by Pauly et al. [45].

Fourteen transgenic lines transformed with the silencing cassette for *Sina* silencing and two for *Sinb* were selected after *Agrobacterium*-mediated transformation. As was already documented in the case of gene silencing [28] as well as transgene expression [29,30], this method should be the method of choice. Segregation of silencing was observed among progeny of all tested T_1_ lines documenting high efficiency of the RNAi silencing process. Similar to silencing of *Pin* genes in wheat [22], both *Sin* genes were silenced at a similar level independent of the silencing cassette used. This feature is not explained by long RNAi trigger size and high homology of *Sina* and *Sinb*. The putative hairpin precursors (hpRNA) from both silencing cassettes did not form short interfering RNA (siRNA) 21–24 nt long, which could repress the expression of both genes.

Selection of silent siblings up to the fourth generation resulted in a uniform level of silencing among T_3_ progeny of most of the sublines. The level of silencing of *Sin* genes measured by qRT-PCR was up to 99%, and was even several percent lower than in the case of *Pin* genes in wheat [22]. The reported expression of other silent genes in wheat relative to the control ranged from 10% to 80% [25-27]. Comparison of expression to other alleles/mutants of *Sin* in rye, *Secale cereale* or triticale genotypes was impossible because of the lack of such studies. This high level of silencing of *Sin* and *Pin* genes might be the result of the main advantage of RNAi technology, which is the possibility to silence all homologous or orthologous genes as well as additional copies of the genes [24,50]. As reported by Wilkinson et al. [51], additional variants of *Pinb* existed in part as a homologous series on the long arm of chromosome 7A. These variants, renamed as *Pinb*-2 series as well as novel variants found were mapped on chromosomes 7D, 7B and 7A [52,53]. Expression of these variants and their impact on grain texture was also reported [54,55]. Triticale shares with wheat two of three genomes, A and B, where all the orthologs to *Sina* and *Sinb* might be silenced. Simultaneous silencing of orthologs or homologs of *Sin* might determine that the level of RNAi silencing would always exceed the decrease of transcript in mutant or even in lack-of-function genotypes.

Silencing of *Sin* genes resulted in low or almost lack of products, secaloindoline a and secaloindoline b. The most unexpected result of *Sin* silencing was the lack of correlation between low expression level of silent genes and increased grain hardness as was observed in wheat [22]. Moreover, correlation coefficients indicated a weak positive relationship between decrease of the transcripts and decrease of the hardness. Taking into account that generally there is no grain hardness variation in rye, our results may suggest that grain hardness in triticale is not related to *Sin* genes and other factors might control this trait in both mentioned species. However, without knowledge about allelic variations of *Sin* genes in both rye and triticale we cannot draw clear conclusions. The hardness index was measured by the SKCS method, which is the current reference method for determination of kernel texture for wheat [56]. The same method was used for grain hardness measurement and classification in 171 triticale lines [14]. Good correlation was also been found between SKCS and PSI (particle size index) method of hardness measurement in barley [57]. The values ranged from 22 to 56 in the silent triticale lines and from 37 to 49 in the control, and the mean values were lower in the silent ones, proving increase of softness. Smaller sample sizes used in our experiment might give some skewed results, however the SKCS data indicate general trend of hardness. Decrease of expression of *Sin* genes was correlated with lower secaloindoline a and secaloindoline b content, influencing the total seed protein content, which was significantly lower compared with control lines. Opposite to triticale, the total seed protein content in silenced lines of wheat was the same as in controls (not published yet). This might indicate that the lack of puroindolines in wheat was offset by other proteins and in triticale not. The range of grain hardness measured using SKCS for 171 hexaploid triticale lines ranged from 8.6 to 84.9 [14]. Opposite to our results, the authors reported that all soft lines contained a high level of friabilin. Consistent with the function of grain hardness *Pin* genes in wheat was also the role of *Hin* genes in barley. The mutation in these genes resulted in an increase in grain hardness [58]. However, in another report low variation in grain hardness of a broad range of barley breeding lines and commercial varieties was documented [57].

The identities of secaloindoline a and secaloindoline b bands on the gel were proved by mass spectrometry and comparison with a protein sequence database using Mascot search engine. Both SINA and SINB from triticale cv. Wanad showed the highest percent of identity with SINA and SINB from soft grain cultivars of *S. cereale*, what might suggest their functionality. The rest of identified puroindolines belong to different species of Triticeae or were identified as cereal-type AAIs and SS protein subfamily – regions existing in the SIN proteins. They are described as mainly present in the seeds of a variety of plants. AAIs play an important role in the natural defenses of plants [41]. Both SIN proteins contain in these regions tryptophan-rich domains, which are highly similar (differ only one amino acid) to regions coded by wild-type of *Pina-D1a* (PuroA) and *Pinb-D1a* (PuroB). The 13-mer of PuroA constitutes the antimicrobial active center of puroindoline a [59]. The corresponding, 12-mer tryptophan-rich domain in puroindoline b was relatively antimicrobially inactive compared to PuroA [60]. It is probable that these highly conserved regions of SIN proteins express the same properties. These data are also consistent with the hypothesis, that positive selection at the *Pina* region of the *Hardness* locus in the tribe Triticeae was congruent with its role as plant defense gene [32]. Besides the effect of puroindolines on the grain hardness determining technological end-uses and their role in plant defense some new biological functions are suggested [45,61]. The results of proteome analysis of soft and hard near-isogenic wheat lines revealed their possible involvement in the storage protein folding machinery affecting the development of wheat endosperm and the formation of the protein matrix [61].

One of the important advantages of RNAi type of silent lines obtained in the process of stable transformation is inheritance of the traits. The T_4_ kernels from the T_3_ generation of triticale plants, which showed an almost equal level of silencing among progeny, might be treated as homozygotes. These selected lines gave a good opportunity for further research concerning other possible functions of *Sin* genes including their influence on the quality, their role in biotic stresses, polar lipids and in the storage protein folding machinery.

## Conclusions

The general effect of RNAi-based silencing of *Sin* genes in hexaploid triticale concerning significant decrease of their transcripts and very low level of both secaloindoline proteins were consistent with that obtained for silencing of *Pin* genes in hexaploid wheat. The unexpected differences occurred on phenotypic level. Silencing of *Sina* and *Sinb* did not determine grain hardness contrary to their orthologs *Pina* and *Pinb* in wheat. Another difference was the total seed protein content in silent lines, which was significantly lower in triticale and similar to control lines in wheat. Since both secaloindoline proteins are present in wild-type, medium-soft phenotype of cv. Wanad and grain hardness remains unchanged in silenced lines with strongly decreased level of both SIN proteins we propose that their biological function does not affect grain texture. Selected lines of triticale and wheat showing siRNA-mediated allele-specific silencing of both *Sin* and *Pin* genes might be useful in further research.

## Methods

### Plant material

Donor plants of the Polish spring triticale (*x Triticosecale* Wittmack) cultivar Wanad were grown under controlled environmental conditions with 18 / 15°C day / night temperatures and 16 h photoperiod. The light intensity was 350 μmol · s^-1^ · m^-2^. Six seeds of each line were seeded into 17 cm × 23 cm × 17 cm pots filled with Aura substrate for sowing and bed out (Hollas sp. z o.o.). Plants were irrigated twice a week and fertilized ones a week with multicomponent soil fertilizer florovit (http://www.florovit.pl) according to producer instructions.

### Vector construction and *Agrobacterium*-mediated transformation

The hpRNA cassettes for silencing of *Sina* and *Sinb* genes were constructed using the RNAi vector pMCG161 (http://www.chromdb.org/rnai/pMCG161.html). The T-DNA of this vector contained the selection gene *bar* under control of the Ubi1 intron promoter and the silencing cassette with two restriction sites separated by a rice waxy intron driven by the CaMV 35S promoter and terminated with OCS3’ terminator. Two hpRNA cassettes were constructed by cloning the full coding sequences of *Pina* or *Pinb* genes in a sense and antisense orientation as described by Gasparis et al. [22]. The 447 bp fragments of both genes were amplified using DNA isolated from *T. aestivum* cv Kontesa. The following primers containing appropriate restriction sites for cloning to the pMCG161 vector were used: for *Pina* (forward) 5′-TTCGGACCGACTAGTATGAAGGCCCTCTTCCTCATA-3′, (reverse) 5′-TTCCTAGGCCCGGGTCACCAGTAATAGCCAATAGTG-3′; for Pinb (forward) 5′-TTCGGACCGACTAGTATGAAGACCTTATTCCTCCTA-3′, (reverse) 5′-TTCCTAGGCCCGGGAGTAATAGCCACTAGGGAACTT-3′. The resulting vectors were pMCG (Pina) for silencing of the *Sina* gene, pMCG (Pinb) for silencing the *Sinb* gene, and the empty pMCG, which is the pMCG161 vector without a silencing cassette. They were electroporated to the *Agrobacterium tumefaciens* strain AGL1 and used either for independent transformation or co-transformation of cv Wanad triticale. The transformation experiments were performed based on our previously described protocols [31].

### PCR amplification

Genomic DNA was isolated from young leaves according to the modified CTAB procedure [62]. PCR reactions were carried out in a 25 μl reaction mixture containing 1 × PCR buffer, 2 mM of MgCl_2_, 0.2 mM of dNTPs, 0.4 μM of each primer, 1 U of Platinum Taq polymerase (Life Technologies), and 120 ng of template DNA. Integration of the silencing cassettes in transgenic plants was examined by amplification of different fragments of T-DNA with five pairs of primers. The pM1,2 and pM3,4 oligos primed the amplification of 902 bp and 829 bp fragments of sense and antisense inserts respectively. Three pairs of qOCS oligos primed the amplification of the OCS3’ terminator fragments of 171 bp (qOCS1,2), 171 bp (qOCS3,4), and 182 bp (qOCS5,6). The sequences of primers and PCR conditions were as described previously [22].

The full coding sequence of the *Sina* gene was amplified using two pairs of specific primers. The first pair were Seca_F (forward) 5′-GGTGTGGCCTCATCTCATCT-3′ and Wpa_R (reverse) 5′-ACCTGGCAGTGGTGGAAATGGT-3′. The second pair were Wpa_F (forward) 5′-CTTCCACCATTTCCACCACTGCCAGGT-3′ and Seca_R (reverse) 5′-AAATGGAAGCTACATCACCAGT-3′. The Seca primers were described previously by Massa and Morris [32] and Wpa primers were designed by us using the program Primer3plus. The full coding sequence of the *Sinb* gene was amplified using primers designed by Lillemo et al. [63]: forward 5′-CATCTATTCATCTCCACCTGC-3′, reverse 5′-GTGACAGTTTATTAGCTAGTC-3′. Annealing temperature for each pair of primers was 58°C and the remaining amplification conditions were as described above.

### RNA isolation and cDNA synthesis

Total RNA was isolated from immature kernels 8, 14, 20, 26 and 32 days after pollination (DAP) using a modified procedure of Prescott and Martin [64]. An additional extraction step with TRI-Reagent (Life Technologies) was performed according to the manufacturer’s protocol. After extraction RNA samples were treated with DNaseI, RNase Free (Roche) to remove the residual genomic DNA. cDNA was synthesized from 1 μg of RNA using RevertAid First Strand cDNA Synthesis Kit (Thermo Scientific).

### Quantitative RT-PCR analysis

26 DAP samples were chosen for qPCR analysis of the relative expression level of *Sina* and *Sinb* genes in silenced transgenic plants. The sequences of the primers were as follows: for *Sina*, qPinA1 (forward) 5′-CTCATAGGACTGCTTGCTCTGGTAG-3′, qPinA2 (reverse) 5′-GATTGACCCCTGGATGATGTTG-3′; for *Sinb*, qPinB1 (forward) 5′-AATGAAGTTGGCGGAGGAGGTG-3′, qPinB2 (reverse) 5′-ATACCTCACCTCGCCAAATGCC-3′; and for 18S rRNA, 18S F (forward) 5′-GTGACGGGTGACGGAGAATT-3′, 18S R (reverse) 5′-GACACTAATGCGCCCGGTAT-3′ [65]. The reaction was carried out in a 15 μl mixture containing 1x Sso Fast EvaGreen Supermix (Bio-Rad), 0.4 μM of each primer, and 1 μl of the template cDNA. The following temperature profile was used: an initial denaturation step of 95°C for 2 min, amplification at 95°C for 25 s, 58°C for 25 s, and 72°C for 25 s, and a melting curve profile of 72–95°C with the temperature rising 1°C at each step and continuous fluorescence measurement. The relative expression level of *Sina* and *Sinb* was calculated according to the ΔΔCt method using 18S rRNA as a normalizer. The values of the three replicates of each sample were used for the calculation. The line transformed with the empty vector pMCG161 was designated as a calibrator sample with its expression value set to 1. The normalized values of the tested samples are expressed as x-fold of 1.

### Extraction of proteins and SDS-PAGE analysis

Secaloindoline proteins were extracted from the water-washed starch fraction of endosperm using the method of Bettge et al. [66] with modifications according to Chang et al. [67]. For each sample an equal amount of starch was used for extraction of proteins.

The SDS-PAGE buffers and gels were prepared according to Laemmli [68]. The stacking gels were 5% T, 2.6% C, and the resolving gels were 15% T, 2.6% C, 1.5 mm thick. 18 × 16 cm gels were resolved in a Hoeffer SE 660 apparatus at 25 mA until the dye reached the bottom edge of the gel. After electrophoresis the gels were silver stained using the protocol of Gromova and Celis [69].

### Identification of protein contents of the sample

SDS PAGE bands (<15 kDa) of SINA and SINB were cut off from the gel and prepared for qualitative mass spectrometry analysis (http://mslab-ibb.pl/en/uslugi). A procedure includes following steps: reduction and alkylation of protein disulfide bonds, followed by tryptic digestion to obtain peptide mixture, liquid chromatography (LC) separation of a sample, MS measurement of peptides and their fragmentation spectra (tandem mass spectrometry) and searching of acquired spectra against a protein sequence database of choice (NCBI, UniProt or customer-supplied database) using Mascot search engine (http://www.matrixscience.com/), followed by validation and formatting of results.

### SKCS, total seed protein and statistical analysis

Grain hardness, weight, moisture, and diameter of kernels were analyzed using the Single-Kernel Characterization System 4100 (Perten Instruments). Each sample contained 35 or 50 kernels and all parameters were calculated for each individual kernel. In total 47 individual plants from T2 and T3 generation were tested: 24 from 12 lines silenced by Pina cassette; 17 from 7 lines silenced by Pinb cassette and 6 from 3 lines transformed with the PMCG without silencing cassette. The grain protein content was determined via near-infrared transmission using a grain analyzer for the same lines (Infratec 1225).

Statistical analysis was performed using Statistica software (StatSoft). The correlation coefficient between the expression level of Sin genes and the grain hardness was calculated in Microsoft Excel.

## Competing interests

The authors declare that they have no competing interests.

## Authors’ contributions

SG carried out most of the experiments: vector construction, genetic transformation, selection and analysis of plants; WO coordinated some experiments (vector construction, analysis of expression) and took part in discussion of the project; ANO have made substantial contributions to conception and design, analysis and interpretation and wrote the manuscript. All authors read and approved the final manuscript.

## Supplementary Material

Additional file 1: Figure S1 Alignment of the *Sina* and *Sinb* sequences of cv. Wanad with others deposited in GenBank by DNASTAR Lasergene software. Click here for file

Additional file 2: Figure S2 Alignment of the *Sina* and *Sinb* sequences of cv. Wanad with *Pina* and *Pinb* by DNASTAR Lasergene software. Click here for file

Additional file 3: Figure S3 Alignment of the SINA and SINB amino acid sequences of cv. Wanad with others puroindoline-like proteins find by Mascot (Table 3). Click here for file
