# Supplementary material for: Sina and Sinb genes in triticale do not determine grain hardness contrary to their orthologs Pina and Pinb in wheat
Source: BMC Plant Biol. 2013 Nov 26;13:190. doi: 10.1186/1471-2229-13-190 (PMC4222565; doi:10.1186/1471-2229-13-190)
Supplement: Additional file 1: Figure S1 — Alignment of the Sina and Sinb sequences of cv. Wanad with others deposited in GenBank by DNASTAR Lasergene software. [file 1471-2229-13-190-S1.pdf]

Sina: Wanad / *Secale cereale*

```
Wanad       ATGAAGGCCTTCTTCTTCGTAGCCCTCCTTGCTCTGGTAGCGAGCACCGC      50
AJ249932.1  .........C.....C..A...GA..G.......................      50
DQ269850.1  ..................................................      50

Wanad       CTTTGCGCAATATAGCGAAGTTG---GCAGTTACGATGTTGCCGGCGGGG      97
AJ249932.1  .......................TTG................T.......     100
DQ269850.1  .......................---........................      97

Wanad       GTGGTGCTCAACAATGCCCTCTAGAGACAAAGCTAAACTCATGCAGGAAT     147
AJ249932.1  ....................G................T............     150
DQ269850.1  ..............................................T...     147

Wanad       TACCTGCTAGAAAGATGCTCAACCATGAAGGATTTCCCGGTGACCTGGCA     197
AJ249932.1  ...........TC..........G.................C.......G     200
DQ269850.1  ..................................................     197

Wanad       GTGGTGGAAATGGTGGAAGGGAGGTTGTCAAGAGCTCCTTGGGGAGTGTT     247
AJ249932.1  T.................................................     250
DQ269850.1  ..................................................     247

Wanad       GCAGCCAGCTCGGCCAAATGCCACCGCAATGCCGCTGCAACATCATCCAG     297
AJ249932.1  ....T.G...........................................     300
DQ269850.1  ......G...........................................     297

Wanad       GGGTCAATCCAAGGCGATCTCGGTGGCATCTTCGGATTTCAGCGTGACCG     347
AJ249932.1  ...............................................T..     350
DQ269850.1  ..................A...............................     347

Wanad       GGCAAGCAAAGTGATACAAGAAGCCAAGAACCTGCCGCCCAAGTGCAACC     397
AJ249932.1  .........................................G........     400
DQ269850.1  ..................................................     397

Wanad       AGGGCCCTGCCTGCAACATCCCCGGCCCTAGTGGCTATTACTGGTGA        444
AJ249932.1  ........C.................A...T................        447
DQ269850.1  .................................T.............        444
```


AJ249932.1 - Gautier,M.F., Cosson,P., Guirao,A., Alary,R. and Joudrier,P. Puroindoline genes are highly conserved in diploid ancestor wheats and related species but absent in tetraploid Triticum species. Plant Sci. 153, 81-91 (2000)

DQ269850.1 - Massa,A.N. and Morris,C.F. Molecular evolution of the puroindoline-a, puroindoline-b, and grain softness protein-1 genes in the tribe Triticeae. J. Mol. Evol. 63 (4), 526-536 (2006)

Sinb: Wanad / *Secale cereale*

```
Wanad      ATGAAGACCTGCTTATTCCTCCTAGCTCTCCTTGCTCTTGTAGCGAGCAC      50
AY667061.1 ..........---.....................................      47
AY667062.1 ..........---.....................................      47
DQ269886.1 ..................................................      50

Wanad      AACCTTGGCGCAATACTCAGAAGTTGGCGGCTGGTACAATGAAGTTGGCG     100
AY667061.1 ..................................................      97
AY667062.1 ..................................................      97
DQ269886.1 ..................................................     100

Wanad      GAGGAGGTGGTGCTCAACAATGCCCGCTGGAGCGGCCGAAGCTAAGCTCT     150
AY667061.1 .....................................C............     147
AY667062.1 .....................................C............     147
DQ269886.1 .....................................C............     150

Wanad      TGCATGGATTACGTGATGGAGCGGTGTTTCACAATGAAGGATTTTCCAGT     200
AY667061.1 ..................................................     197
AY667062.1 ..................................................     197
DQ269886.1 ..................................................     200

Wanad      CACTTGGCCCACGAGATGGTGGAAGGGCGGTTGTGAGCACGAGGTTCGGG     250
AY667061.1 ..................................................     247
AY667062.1 ..................................................     247
DQ269886.1 ..................................................     250

Wanad      AGAAGTGTTGCAATCAGCTGAGTCAGATAGCACCACAGTGTCGCTGCGAT     300
AY667061.1 ..................................................     297
AY667062.1 ..................................................     297
DQ269886.1 ..................................................     300

Wanad      TCTATCCGAGGAATGATCCAAGGCAAGTTCGGAGGCTTCTTCGGCATTTG     350
AY667061.1 ..................................................     347
AY667062.1 .....................A............................     347
DQ269886.1 .....................A............................     350

Wanad      GCGAGGTGATGTATTCAAACAAACTCAAAGGGCCCAGAGCCTCCCCTCGA     400
AY667061.1 ..................................................     397
AY667062.1 ...........................G......................     397
DQ269886.1 ...........................G......................     400

Wanad      AGTGCAACATGGGAGCCGACTGCAAGTTCCCTGGTGGCTATTACTGGTGA     450
AY667061.1 ..................................................     447
AY667062.1 ................................C.................     447
DQ269886.1 ................................C.................     450
```


AY667061.1 - Simeone,M.C. and Lafiandra,D. Isolation and characterization of friabilin genes in rye. J. Cereal Sci. 41 (1), 115-122 (2005)

AY667062.1 - Simeone,M.C. and Lafiandra,D. Isolation and characterization of friabilin genes in rye. J. Cereal Sci. 41 (1), 115-122 (2005)

DQ269886.1 - Massa,A.N. and Morris,C.F. Molecular evolution of the puroindoline-a, puroindoline-b, and grain softness protein-1 genes in the tribe Triticeae. J. Mol. Evol. 63 (4), 526-536 (2006)

Figure S1. Alignment of the *Sina* and *Sinb* sequences of cv. Wanad with others deposited in GenBank by DNASTAR Lasergene software.
