# Supplementary material for: Sina and Sinb genes in triticale do not determine grain hardness contrary to their orthologs Pina and Pinb in wheat
Source: BMC Plant Biol. 2013 Nov 26;13:190. doi: 10.1186/1471-2229-13-190 (PMC4222565; doi:10.1186/1471-2229-13-190)
Supplement: Additional file 2: Figure S2 — Alignment of the Sina and Sinb sequences of cv. Wanad with Pina and Pinb by DNASTAR Lasergene software. [file 1471-2229-13-190-S2.pdf]

Pina-D1a / Sina Wanad (seq1 - KC784350)

```
850-1296 ATGAAGGCCCTCTTCCTCATAGGACTGCTTGCTCTGGTAGCGAGCACCGC          50
Seq1     .........T.....T..G...CC..C.......................          50

850-1296 CTTTGCGCAATATAGCGAAGTTGTTGGCAGTTACGATGTTGCTGGCGGGG         100
Seq1     .......................---................C.......          97

850-1296 GTGGTGCTCAACAATGCCCTGTAGAGACAAAGCTAAATTCATGCAGGAAT         150
Seq1     ....................C................C............         147

850-1296 TACCTGCTAGATCGATGCTCAACGATGAAGGATTTCCCGGTCACCTGGCG         200
Seq1     ...........AA..........C.................G.......A         197

850-1296 TTGGTGGAAATGGTGGAAGGGAGGTTGTCAAGAGCTCCTTGGGGAGTGTT         250
Seq1     G.................................................         247

850-1296 GCAGTCGGCTCGGCCAAATGCCACCGCAATGCCGCTGCAACATCATCCAG         300
Seq1     ....C.A...........................................         297

850-1296 GGGTCAATCCAAGGCGATCTCGGTGGCATCTTCGGATTTCAGCGTGATCG         350
Seq1     ...............................................C..         347

850-1296 GGCAAGCAAAGTGATACAAGAAGCCAAGAACCTGCCGCCCAGGTGCAACC         400
Seq1     .........................................A........         397

850-1296 AGGGCCCTCCCTGCAACATCCCCGGCACTATTGGCTATTACTGGTGA            447
Seq1     ........G.................C...G................            444
```

Pinb-D1a / Sinb Wanad (seq2 - KC784351)

```
274-720 ATGAAGACCT---TATTCCTCCTAGCTCTCCTTGCTCTTGTAGCGAGCAC          47
Seq2    ..........GCT.....................................          50

274-720 AACCTTCGCGCAATACTCAGAAGTTGGCGGCTGGTACAATGAAGTTGGCG          97
Seq2    ......G...........................................         100

274-720 GAGGAGGTGGTTCTCAACAATGTCCGCAGGAGCGGCCGAAGCTAAGCTCT         147
Seq2    ...........G..........C....T......................         150

274-720 TGCAAGGATTACGTGATGGAGCGATGTTTCACAATGAAGGATTTTCCAGT         197
Seq2    ....T..................G..........................         200

274-720 CACCTGGCCCACAAAATGGTGGAAGGGCGGCTGTGAGCATGAGGTTCGGG         247
Seq2    ...T........G.G...............T........C..........         250

274-720 AGAAGTGCTGCAAGCAGCTGAGCCAGATAGCACCACAATGTCGCTGTGAT         297
Seq2    .......T.....T........T..............G........C...         300

274-720 TCTATCCGGCGAGTGATCCAAGGCAGGCTCGGTGGCTTCTTGGGCATTTG         347
Seq2    ........AG..A............A.T....A........C........         350

274-720 GCGAGGTGAGGTATTCAAACAACTTCAGAGGGCCCAGAGCCTCCCCTCAA         397
Seq2    .........T............AC...A....................G.         400

274-720 AGTGCAACATGGGCGCCGACTGCAAGTTCCCTAGTGGCTATTACTGGTGA         447
Seq2    .............A..................G.................         450
```

Figure S2. Alignment of the *Sina* and *Sinb* sequences of cv. Wanad with *Pina* and *Pinb* by DNASTAR Lasergene software.
