# Supplementary material for: Sina and Sinb genes in triticale do not determine grain hardness contrary to their orthologs Pina and Pinb in wheat
Source: BMC Plant Biol. 2013 Nov 26;13:190. doi: 10.1186/1471-2229-13-190 (PMC4222565; doi:10.1186/1471-2229-13-190)
Supplement: Additional file 3: Figure S3 — Alignment of the SINA and SINB amino acid sequences of cv. Wanad with others puroindoline-like proteins find by Mascot (Table 3). [file 1471-2229-13-190-S3.pdf]

SINA cv. Wanad

```
AGO65289.1  MKAFFFVALLALVASTAFAQYSEVG-SYDVAGGGGAQQCPLETKLNSCRNYLLERCSTMK 59
ABB88759.1  MKAFFFVALLALVASTAFAQYSEVG-SYDVAGGGGAQQCPLETKLNSCSNYLLERCSTMK 59
AER62832.1  MKAFFLIGLLALVASTAFAQYREVVGSYDVAGXGGAQQCPLDTKLNSCRNYLLDRCITMK 60
ABD72477.1  MKAIFLIGLLALVASTAFAQYSEVVGSYDVAGGGGAQQCPVETKLNSCRNYLLDRCSTMK 60
ABB52757.1  ----------------------------DVAGGGGAQQCPIETKLNSCRNYLLDRCSTMK 32

AGO65289.1  DFPVTWQWWKWWKGGCQELLGECCSQLGQMPPQCRCNIIQGSIQGDLGGIFGFQRDRASK 119
ABB88759.1  DFPVTWQWWKWWKGGCQELLGECCSRLGQMPPQCRCNIIQGSIQGDIGGIFGFQRDRASK 119
AER62832.1  DFPXTWXWWKWWKGGCQELLGECCSRLGQMPPQCRCNIIQGSIQGDLGGIFGFQRDRAGK 120
ABD72477.1  DFPVTWRWWKWWKGGCQELLGECCSRLGQMPPQCRCNIIQGSIQGDLGGIFGFQRDRASK 120
ABB52757.1  DFPVTWRWWKWWKGGCLELLGECCSRLGQMPPQCRCNIIQGSIQGDLGGIFGFQRDRASK 92

AGO65289.1  VIQEAKNLPPKCNQGPACNIPGPSGYYW                                 147
ABB88759.1  VIQEAKNLPPKCNQGPACNIPGPSVYYW                                 147
AER62832.1  VIQEAKNLPPKCNQGPPCDIPSTSGYYW                                 148
ABD72477.1  VIQEAKNLPPRCNQGPPCNIPGTIGYYW                                 148
ABB52757.1  VIQEAKNLPPRCNQGPPCNIPGTIGYYW                                 120
```

AGO65289.1 – secaloindoline a – (x *Triticosecale* Wittmack) cv. Wanad
ABB88759.1 – puroindoline a – *Secale cereale* cv. Galma
AER62832.1 – puroindoline a – *Elymus spicatus* (synonyms: *Pseudoroegneria spica* and *Agropyron spicatum*)
ABD72477.1 – puroindoline a – *Triticum aestivum* cv. Chinese Spring
ABB52757.1 – puroindoline a – *Aegilops sharonensis*

Sequence Distances of SINA.meg ClustalW (Slow/Accurate, Gonnet)

Percent Similarity in upper triangle
Percent Divergence in lower triangle

Percent Identity

	1	2	3	4	5		
**1**		97.3	88.4	91.2	92.5	**1**	AGO65289.1
**2**	2.8		87.1	89.8	90.8	**2**	ABB88759.1
**3**	12.6	14.2		91.2	90.0	**3**	AER62832.1
**4**	9.4	11.0	9.4		98.3	**4**	ABD72477.1
**5**	7.9	9.8	10.8	1.7		**5**	ABB52757.1
	**1**	**2**	**3**	**4**	**5**		

Region: 36..135

```
1 mkafffvall alvastafaq ysevgsydva ggggaqqcpl etklnscrny llercstmkd
61 fpvtwqwwkw wkggcqellg eccsqlgqmp pqcrcniiqg siqgdlggif gfqrdraskv
121 iqeaknlppk cnqgpacnip gpsgyyw
```

Region_name="AAI_SS": Alpha-Amylase Inhibitors (AAIs) and Seed Storage (SS) Protein subfamily; composed of cereal-type AAIs and SS proteins.

SINB cv. Wanad

```
AGO65290.1  MKTCLFLLALLALVASTTLAQYSEVGGWYN-EVGGGGGAQQCPLERPKLSSCMDYVMERC 59
AAT76524.1  MKT-LFLLALLALVASTTLAQYSEVGGWYN-EVGGGGGAQQCPLERPKLSSCMDYVMERC 58
ADH94993.1  MKT-FFLLALLALVAGTTFAQYSEVGGWFHNEAGAGGGSQQCPLERPKLSSCKDYVMERC 59
ABB88792.1  MKT-LFLLPLLALVASTAFAQYSEVGGWYN-EVGAGGGSQQCPLERPKLSSCKYYVMERC 58
ABD72479.1  MKT-LFLLALLALVASTTFAQYSEVGGWYN-EVGGGGGSQQCPQERPKLSSCKDYVMERC 58
BAK64217.1  MKT-LFLLALLALVASTTLAQYS-VGGGYN-EVGGGGGSQQCPQERPNLSSCKDYVMERC 57

AGO65290.1  FTMKDFPVTWPTRWWKGGCEHEVREKCCNQLSQIAPQCRCDSIRGMIQGKFGGFFGIWRG 119
AAT76524.1  FTMKDFPVTWPTRWWKGGCEHEVREKCCNQLSQIAPQCRCDSIRGMIQSKFGGFFGIWRG 118
ADH94993.1  FTMRDFPVTWPTRWWKGGCEHEVREKCCKQLSQIAPQCRCDSIRGMMQGKLGGFFGIWRG 119
ABB88792.1  LTMKDFPVTWPTRWWKGGCEHEVREKCCQQLSQIAPQCRCDSIRGMIQSKLGGFFGIWRG 118
ABD72479.1  FTMKDFPVTWPTKWWKGGCEHEVREKCCKQLSQIAPQCRCDSIRRVIQGRLGGFLGIWRG 118
BAK64217.1  FTMKDFPVTWPTRWWKGGCEHEVRDKCCQQLSQIAPQCRCDSIRRVIQSKLGGIFGIWRG 117

AGO65290.1  DVFKQTQRAQSLPSKCNMGADCKFPGGYYW                               149
AAT76524.1  DVFKQTQRAQSLPSKCNMGADCKFPRGYYW                               148
ADH94993.1  DVFKQIQRAQSLPSKCNMGADCKLPIGYYW                               149
ABB88792.1  DVFKQIQRAQSLPSKCNMGADCKFPSAYYW                               148
ABD72479.1  EVFKQLQRAQSLPSKCNMGADCKFPSGYYW                               148
BAK64217.1  EVYKQIQRAQSLPSKCNMGADCKFPSGYYW                               147
```

AGO65290.1 – secaloindoline b – (x *Triticosecale* Wittmack) cv. Wanad
AAT76524.1 – puroindoline b – *Secale cereale* cv. Imperial
ADH94993.1 – puroindoline b – *Aegilops comosa*
ABB88792.1 – puroindoline b – *Aegilops umbellulata*
ABD72479.1 – puroindoline b – *Triticum aestivum* cv. Chinese Spring
BAK64217.1 – hordoindoline b – *Hordeum brachynatherum* subsp. Californicum

Sequence Distances of SINB.meg ClustalW (Slow/Accurate, Gonnet)
Percent Similarity in upper triangle
Percent Divergence in lower triangle

Percent Identity

	1	2	3	4	5	6		
**1**		98.6	89.2	90.5	90.5	89.1	**1**	AGO65290.1
**2**	1.4		88.5	91.2	89.9	89.8	**2**	AAT76524.1
**3**	11.7	12.5		89.2	87.8	85.0	**3**	ADH94993.1
**4**	10.1	9.4	11.7		89.2	89.1	**4**	ABB88792.1
**5**	10.1	10.9	13.3	11.7		91.8	**5**	ABD72479.1
**6**	11.8	11.0	16.7	11.8	8.7		**6**	BAK64217.1
	**1**	**2**	**3**	**4**	**5**	**6**		

Region 39..137

```
1 mktclfllal lalvasttla qysevggwyn evgggggaqq cplerpklss cmdyvmercf
61 tmkdfpvtwp trwwkggceh evrekccnql sqiapqcrcd sirgmiqgkf ggffgiwrgd
121 vfkqtqraqs lpskcnmgad ckfpggyyw
```

Region_name="AAI_SS": Alpha-Amylase Inhibitors (AAIs) and Seed Storage (SS) Protein subfamily; composed of cereal-type AAIs and SS proteins.

Figure S3. Alignment of the SINA and SINB amino acid sequences of cv. Wanad with others puroindoline-like proteins find by Mascot (Table 3).
